# The effect of *Crocus sativus* L. (saffron) herbal tea on happiness in postmenopausal women: a randomized controlled trial

**DOI:** 10.1186/s12906-023-04014-8

**Published:** 2023-06-01

**Authors:** Hamed Delam, Zahra Keshtkaran, Nasrin Shokrpour, Ahmadreza Eidi, Mohammad-Rafi Bazrafshan

**Affiliations:** 1 Student Research Committee, Larestan University of Medical Sciences, Larestan, Iran; 2 Imam Reza Teaching Hospital, Larestan University of Medical Sciences, Larestan, Iran; 3 Community Based Psychiatric Care Research Center, Department of Nursing, School of Nursing and Midwifery, Shiraz University of Medical Sciences, Shiraz, Iran; 4 English Department, Shiraz University of Medical Sciences, Shiraz, Iran; 5 Department of Nursing, School of Nursing, Larestan University of Medical Sciences, Larestan, Iran

**Keywords:** Crocus, Herbal tea, Happiness, Menopause, Depression

## Abstract

**Background:**

Evidence suggests that menopause can be associated with a variety of negative psychological changes such as depression and anxiety, and improving the mental health status of women during menopause is one of the important priorities and challenges of the health system. The aim of this study was to determine the effect of saffron (*Crocus sativus* L., Iridaceae) herbal tea on happiness in postmenopausal women.

**Methods:**

In this randomized clinical trial which was conducted in 2021, 72 postmenopausal women were enrolled and divided into intervention and control groups. The randomization blocks method was used for random allocation, and the Oxford Happiness Questionnaire was utilized to measure the scores. The intervention included the use of 30 mg of dried stigmas of the saffron plant, which was boiled once (in the morning, in 300 ml of boiling water for 10–15 min) and consumed with white rock candy as one cup of saffron tea daily. To compare the trend of changes and after removing the effect of other variables, generalized estimating equation (GEE) was used.

**Results:**

There was no significant difference between the intervention and control groups in any of the quantitative and qualitative characteristics (*p* > 0.05). The results of paired samples t-test showed that the happiness mean score in the intervention group increased significantly (*p* < 0.001) from 42.93 ± 8.54 to 61.58 ± 8.24, while in the control group, there was no significant difference between the happiness mean score at the beginning and end of the study (*p* = 0.861). Also, after applying the treatment program in the intervention group, there was a significant difference between the two groups in terms of the happiness mean scores (*p* < 0.001).

**Conclusion:**

Saffron herbal tea had a positive effect on reducing depression and increasing the happiness score; thus, it is recommended that it should be used as a complementary treatment in consultation with the treating physician.

**Trial registration:**

The present study was registered with the code of IRCT20210403050818N1 (Registration date: 09/04/2021) in the Iranian Registry of Clinical Trials. It was also approved by the Ethics Committee of Larestan University of Medical Sciences (Approval ID: IR.LARUMS.REC.1399.017).

## Background

Women are regarded as the foundation of family health; in addition to managing the health of the family members, they are the main role model of educating and promoting a healthy lifestyle for future generations. Menopause is one of the natural processes in a female’s life, the most prominent feature of which is the end of fertility and menstruation [1]. Menopause is a natural physiological process in older women in which the number of primary follicles in the ovaries rapidly decreases and ovulation does not occur, resulting in decreased estrogen production and cessation of menstruation for at least 12 months [2]. In general, normal menopause occurs in Western societies due to the erosion of ovarian follicles at the age of about 51 years [3, 4]. The age of normal menopause in women in developed countries is from 50 to 52 years [5, 6], while in less developed countries it is 3–4 years earlier [7]. In Iran, the mean age of menopause is about 47.8 years [1]. Changes in family roles along with the feeling of aging might lead to psychological symptoms. Also, following the psychosocial changes in this period of life, there is considerable evidence that reproductive hormones, especially estrogen, may have an effect on mood [8]. Evidence suggests that menopause can be associated with a variety of negative psychological changes, such as depression and anxiety [9]. On the other hand, happiness is a concept that is essential for physical and mental health and is defined as having complete, permanent, and convincing satisfaction in life. Happiness is an important factor in human life, during which a person will always feel good about himself and other people. Happy people will usually have higher productivity, higher levels of hopes, academic achievement, and a better quality of life [10]. According to some evidence, negative mood changes in postmenopausal women are associated with the occurrence of consequences such as disability [11], significant interpersonal-occupational and occupational dysfunction, as well as the high cost of health care [9]. Therefore, improving the mental health status of women during menopause is one of the important priorities and challenges of the health system. Among various therapies used to improve the individuals’ mental state, non-pharmacological methods have a special value in new therapeutic approaches [12]. Non-pharmacological or complementary medicine methods such as music therapy, hypnosis, acupuncture, yoga, massage therapy, touch therapy, aromatherapy, and the use of herbal teas often have few side effects and few risks and are used alone or in combination with other methods to improve the mental state of individuals [13]. In the United States, about 40 percent of people use different methods of complementary medicine, some of which are within the scope of nursing work and can be part of the care programs performed by nurses [14]. For this reason, nurses are trying to use complementary medicine treatments to improve the patients' peace and recovery and ultimately their mental health [15]. Saffron (*Crocus sativus* L.) is a small perennial plant belonging to the *Iridaceae* family that is cultivated in many countries including Iran, Turkey, Spain, and Afghanistan. Saffron is used as a medicinal plant to enhance the human health, especially in Asia, and its main components include *crocin*, *picrocrocin,* and *safranal* [16]. In a review study that examined the pharmacological effects of saffron, it was found that *crocin* in saffron inhibited the reabsorption of dopamine and norepinephrine; also, *safranal* prevented the reabsorption of serotonin, both playing a role in the antidepressant and stimulant function of saffron. [17]. The results of a laboratory study conducted on mice showed that in general, saffron significantly reduced the symptoms of depression in mice and partially restored the hippocampus nerve damage. Moreover, the antidepressant mechanism of saffron primarily depended on the regulation of the MAPK-CREB1-BDNF signaling pathway [18].

Another animal study showed that intraperitoneal administration of alcoholic saffron extract with doses of 200 and 800 mg/kg and aqueous extracts of saffron with doses of 160 and 320 mg/kg significantly reduced immobility time compared to normal saline.

In the open box test, saffron aqueous extract, unlike the alcoholic extract, with doses of 160 and 320 mg/kg, significantly reduced overall movement compared to normal saline. A decrease in the time of remaining motionless in the swimming test was observed with the effective substances of saffron, i.e. *safranal* and *crocin*. The duration of swimming increased in the rats which received fluoxetine and *safranal*, but no difference between different doses was seen in the groups which received *crocin*. Different doses of *safranal* and *crocin* increased the rise time almost equally, which were significantly different from normal saline. *Crocin* increased the stereotypic movements and *safranal* decreased the overall movement. Other anti-depressant mechanisms such as monoamine oxidase enzyme inhibition can play a role in this activity. Oxidase of this study shows that probably the antidepressant effect of saffron stigma is at least partly through *crocin* and *safranal*. *Crocin,* as a water-soluble compound of saffron, and *safranal,* as a fat-soluble compound, act with two different mechanisms. *Crocin* is probably effective on the dopaminergic system and inhibits epinephrine and *safranal* light reabsorption on the serotonergic system [19].

A study designed with the aim of the effect of saffron supplement and resistance training on the markers involved in the level of depression and happiness in untrained young men showed that 6 weeks of saffron supplementation along with resistance training improved the level of happiness, serotonin, dopamine, beta endorphin, anandamide and 2-arachidonoylglycerol [20]. Also, in a clinical trial study conducted by Jackson et al. (2021) with the aim of investigating the effects of saffron extract on the mental health of people with subclinical feelings of low mood and anxiety and/or stress who were aged 15 to 57 years, 56 men and women were divided into placebo groups (36 people) and those who had received saffron extract (37 people); the intervention group people received 30 mg of saffron extract for 8 weeks. The findings of the study showed that there was a reduction in depression, anxiety, and stress scores in the intervention group [21].

Given the high number of postmenopausal women, the possibility of unpleasant mood changes among this group due to menopausal complications, the need to improve the mental state of this group, the responsibility of nurses in assessing the mental state of this group of people, the need to implement appropriate measures, and lack of a similar study on the effect of saffron herbal tea on the happiness of postmenopausal women, researchers decided to conduct a study to determine the effect of saffron tea on the postmenopausal women’s happiness. Hopefully, the results of this study will be an effective step in recognizing complementary and alternative medicine treatments, especially the use of herbal teas, in the nursing community, and can alleviate the patients' pain.

## Methods

### Study type

The present study was a randomized controlled trial study conducted in 2021 in Iran.

### Study population

The participants in this study consisted of all postmenopausal women who referred to community health centers affiliated to Larestan University of Medical Sciences in 2021.

### Sample size

To determine the sample size using a similar study [22] and according to the formula of comparing the two means, taking into account the error level of 1%, power 90%, 72 people (36 people in each group) were calculated.

### Sampling

First, the list of community health centers affiliated to Larestan University of Medical Sciences, to which postmenopausal women referred for health services, was extracted. In the next step, the name of each center was placed in a separate envelope and by simple random sampling, the envelope was selected.

### Random allocation

For allocation of individuals to the intervention and control groups, the randomized blocks method was used. According to the total sample size (*n* = 72), six blocks with 12 cells were used. Each block was randomly arranged using the letters A and B (there were 6 A cells and 6 B cells in each block). In the next step, a dice was used to start completing the blocks. This means that at first, by rolling the dice (in the present study, number 3), people entered the study from block 3 and the houses began to be completed in order; finally, with the completion of all 72 cells, 36 women in the intervention group and 36 in the control group were enrolled. It should be noted that according to the previous agreement, each person in cell B was selected for the intervention group and those in cell A for the control group.

### Blinding

In the present research, it was not possible to blind the study participants.

### Inclusion criteria

- Willingness to participate in the study.
- Not suffering from acute psychosis, chronic and debilitating diseases, and cognitive diseases such as dementia.
- Not being under similar treatment recently.
- Not being familiar with the Persian language.
- Not participating in other treatment programs that interfere with the present study.
- Being able to swallow and having no oral or digestive problems that interfere with drinking.
- Lack of menstruation for at least 12 months.

### Exclusion criteria

- Occurrence of any social and family crisis during the study.
- Hospitalization or acute and chronic illness that interferes with research.
- History of allergy to herbal medicines.
- Unwillingness to continue participating in the research.
- Addiction to drugs and alcohol and painkillers.
- Consumption of psychiatric drugs.
- Infection of the participant or her family members with COVID-19.

### Questionnaire

In the present study, two demographic information and happiness questionnaires were used. To complete the questionnaires, two assistant researchers distributed the questionnaires among the participants. Demographic information questionnaire included questions on age, marital status, level of education, and menopausal age.

Happiness Questionnaire: The Oxford Happiness Questionnaire was developed in 1990 by revising the Beck Depression Scale. This scale has 29 items that are graded based on a four-point scale ranging from 1 to 4. Therefore, the minimum score of each subject is 29 and the maximum score 116. The higher the subject's score, the higher his/her happiness, and vice versa [23]. Argyle, Martin and Crossland (1989) obtained 90% alpha coefficient [24] and Francis, Brown, Lester, and Philipchalk (1998) reported Cronbach's alpha of 92% [25]. In another study conducted by Alipour and Noorbala (1999) in a sample of 101 students of universities in Tehran, the internal consistency coefficient for men and women was 94% and 90%, respectively [26].

If willing to cooperate, postmenopausal women were randomly divided into two intervention groups: the group which received saffron herbal tea and control group. Postmenopausal women in each group were then assessed for their happiness using a questionnaire.

### Intervention

The intervention was the use of 30 mg dried stigmas of the saffron plant (Mostafavi company, Iran. This company is one of the largest producers of saffron in Iran and the world), which was boiled once (in the morning) (in 300 ml of boiling water for 10–15 min); one cup of saffron tea was consumed daily with white rock candy [27–29]. The study participants were instructed to use herbal tea completely and avoid using other medicinal plants at the same time, according to the previous agreement and the individual's consent. According to previous research, the duration of this intervention was 6 weeks [20, 30]. Although the total duration of the intervention was considered to be 6 weeks, at the end of the 2nd and 4th weeks, the results of the saffron infusion were determined. The subjects in the intervention group received the assigned number of their drinks (30 mg teabags) for the entire study period (6 weeks) as soon as they entered the study. Subjects in this group were called daily (up to 6 weeks), and the process of saffron consumption was evaluated. Up to a maximum of one session, non-consumption of saffron herbal tea by the intervention group was allowed.

### Control group

In the present study, lukewarm water and white rock candy were considered for the control group, and the subjects in this group were asked to consume it one glass daily. A similar study that measured the effect of lavender tea on depression used lukewarm water and white rock candy for the control group [22]. The white rock candy was used along with lukewarm water only to encourage the control group to cooperate in the project. The white rock candy which was made of heated sugar was devoid of any effective substances related to depression and happiness. On the other hand, for the intervention group, white candy was used to make the two intervention and control groups completely similar as to this factor and neutralize its possible role, although very weak, in changing the happiness score.

### Side effects

According to the results of previous studies, the maximum safe daily dose of saffron is 1.5 g, so it was expected that the dose in the present study did not cause side effects [17]. In the current study, people prescribed 30 mg of saffron daily, which is insignificant compared to the maximum dose, and it seems that the side effect related to saffron does not threaten people. Also, according to Drugs.com, toxic effects have been reported for doses of 5 g, while doses of 10 to 20 g may be fatal [31].

### Ethical considerations

The present study was registered with the code of IRCT20210403050818N1 (Registration date: 09/04/2021) in the Iranian Registry of Clinical Trials. It was also approved by the Ethics Committee of Larestan University of Medical Sciences (Approval ID: IR.LARUMS.REC.1399.017). After assigning the participants to the two groups of intervention and control, informed consent was obtained from them and in all stages of the study, and the participants were ensured that their information would remain confidential.

### Statistical methods

After completing the questionnaires by the samples, the data were entered into SPSS software, version 25. Fisher's exact test was used to measure the relationship between qualitative variables and independent sample t-test was used to compare the mean happiness scores in the intervention and control groups. Also, the comparison of the mean happiness score in the stage before and after the intervention in each group was performed using paired sample t-test. To compare the trend of changes and after removing the effect of other variables, generalized estimating equation (GEE) was used. A statistical significance level of 0.05 was considered.

## Results

**Fig. 1:**
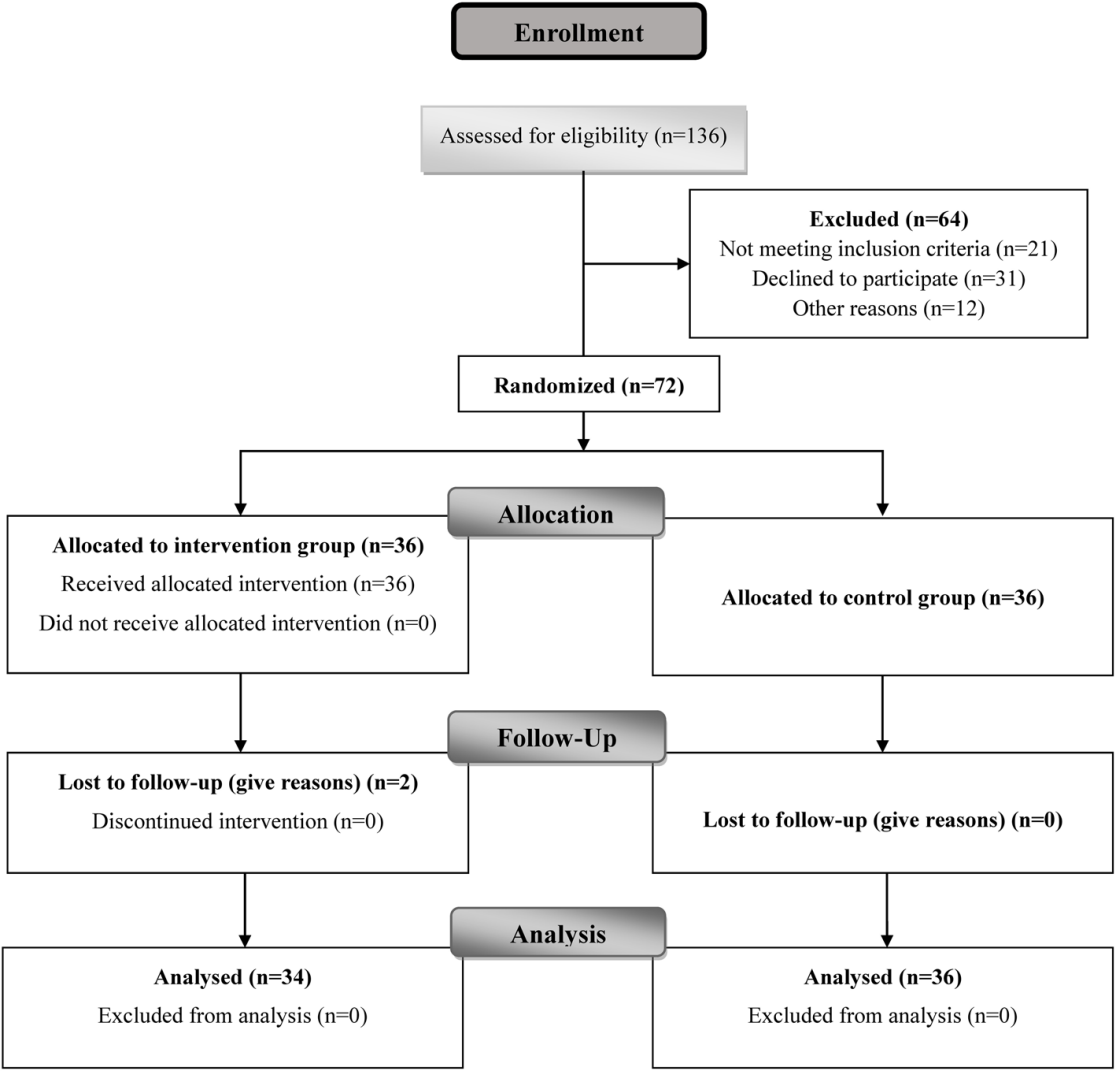
Study protocol

Out of 72 participants in the study, 36 were in the intervention group and 36 in the control group (Fig. 1). The mean age of the subjects in the intervention and control groups was 53.75 ± 3.87 and 53.13 ± 3.91 years, respectively. Also, the mean age at menopause in the intervention and control groups was 47.52 ± 0.0.99 and 47.27 ± 0.91 years, respectively. Most of the women in the study were married and had a non-academic level of education (elementary, high school and diploma). There was no significant difference between the intervention and control groups in any of the quantitative and qualitative characteristics (*p* > 0.05) (Table 1).

**Table 1:** Comparison of quantitative and qualitative characteristics of the participants in the study by the intervention and control groups

Variable	Intervention group	Control group	*P*-value
**Age**, (year), Mean ± SD^a^	53.75 ± 3.87	53.13 ± 3.91	0.508^**^
**Menopausal age**, (year), Mean ± SD^a^	47.52 ± .0.99	47.27 ± 0.91	0.272^**^
**Marital status**, n (%)			
Single	6 (60.0)	4 (40.0)	0.735^***^
Married	30 (48.4)	32 (51.6)	
**Education level**, n (%)			
Non academic	31 (48.4)	33 (51.6)	0.355^***^
Academic	5 (62.5)	3 (37.5)	

^**^Results by independent sample t test

^***^Results by Fisher's Exact Test

^a^Standard Deviation

The results of paired sample t-test showed that the mean happiness score in the intervention group increased significantly (*p* < 0.001) from 42.93 ± 8.54 to 61.58 ± 8.24, while in the control group, there was no significant difference between the mean happiness score at the beginning and end of the study (*p* = 0.861). Also, the comparison of happiness scores in the intervention and control groups at the beginning of the study did not show a significant difference (*p* = 0.148); however, after applying the treatment program in the intervention group, there was a significant difference between the two groups in terms of mean happiness scores (*p* < 0.001) (Table 2).

**Table 2:** Comparison of the mean happiness score before and after the intervention in the intervention and control groups

Groups	Before intervention (Mean ± SD^a^)	After intervention (Mean ± SD^a^)	*P*-value^**^
Intervention	42.93 ± 8.54	61.58 ± 8.24	< 0.001
Control	43.11 ± 6.81	42.75 ± 10.23	0.861
*p*-value^***^	0.148	< 0.001	

^**^Results by Paired sample t test

^***^Results by independent sample t test

^a^Standard Deviation

**Fig. 2:**
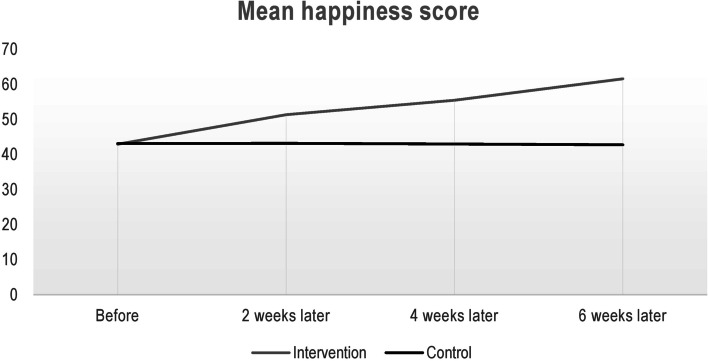
Comparison of changes in the happiness score in the intervention and control groups

Table 3 contains information about the mean (standard deviation) of the investigated outcome and their comparison using the GEE model. As can be seen in this Table and Fig. 2, in the intervention group, the trend of changes was increasing (*p* < 0.001), but in the control group, the trend was constant (*p* = 0.861). In total, the observed difference between the mean score of happiness in the two groups was statistically significant (*p* < 0.001). In this regard, comparison of the happiness scores in the two groups after controlling the effect of demographic variables in the GEE model revealed that there was a statistically significant difference between the two groups (*p* < 0.001).

**Table 3:** Mean (standard deviation) happiness score in the two intervention and control groups and their comparison using GEE model

Outcome	Groups	Time				Effect	
		**Before**	**2 weeks later**	**4 weeks later**	**6 weeks later**	**Time (within group)**	**Between group**^**a**^
Happiness score	Intervention	42.93 ± 8.54	51.36 ± 6.18	55.48 ± 6.10	61.58 ± 8.24	< 0.001	< 0.001
	Control	43.11 ± 6.81	43.18 ± 5.55	42.96 ± 7.89	42.75 ± 10.23	0.861	

^a^After adjustment for demographic variables in GEE model

## Discussion

From the past to the present, saffron and its main derivatives, as well as organoleptic properties, are commonly used in traditional medicine due to their numerous therapeutic properties such as anti-anxiety, anti-depressant, and anti-seizure effects [32]. Complementary therapy has been considered today and many patients use complementary therapies to treat their depression [22]. On the other hand, the use of herbal teas for the treatment of chronic diseases has been considered because of its cost**-**effectiveness [33].

In the present study, saffron herbal tea was used for postmenopausal women. The results of the study showed that saffron herbal tea significantly increased the mean score of happiness in postmenopausal women, and there was a statistically significant difference between the intervention and control groups. In other words, the results showed that saffron herbal tea could have a positive effect on the participants' happiness, which was similar to the results of previous studies.

The findings of Shahmansouri et al.’s [34] study showed that short-term consumption of saffron could be as effective as fluoxetine in improving depression symptoms. In a study conducted by Kashani et al. [35] in Iran on 60 women with postmenopausal hot flashes with the aim of evaluating the effectiveness and safety of saffron capsules in the treatment of major depressive disorder associated with postmenopausal hot flashes, it was found that saffron was a safe and effective treatment to improve hot flashes and depressive symptoms in healthy postmenopausal women. On the other hand, saffron can be a good option in treating such individuals due to fewer side effects. Another study in Iran was conducted by Ghorbani et al. [17] to determine the effect of saffron capsule on happiness in postmenopausal women; it was found that there was a significant difference between the mean happiness score in the intervention group and the control group after consuming saffron capsules, so that the intervention group had a higher happiness score than the controls. A similar study showed that saffron petals were effective in treating mild to moderate depression; in this double-blind clinical trial study, the intervention group received one capsule containing 30 mg of saffron daily [36]. The results of a meta-analysis study showed that saffron supplementation can improve depression symptoms in adults with major depressive disorder [37]. Also, the results of an umbrella meta-analysis published in 2022 showed that the consumption of saffron might help reduce depression, but it could not be considered as a single treatment approach for the treatment of depression [38].

A study on the comparison of the effects of saffron and Imipramine in the treatment of mild to moderate depression showed that a 10 mg dose of saffron capsule had the same effect in the treatment of mild to moderate depression as imipramine. In addition, this study showed that Imipramine had anticholinergic effects such as dry mouth [39]. It seems that the effective substances of saffron exert their anti-depressant effects on the body by modulating different neurotransmitter systems in the brain, including dopamine, serotonin, glutamate and norepinephrine [40].

Human and animal studies have shown that saffron and its compounds have a therapeutic effect on mild to moderate depression, which seems to be due to interaction with serotonin and noradrenaline; however, more clinical and laboratory studies are recommended to find a more precise mechanism of action [16]. In this regard, a study reported that saffron significantly reduced homocysteine levels in both sexes and led to a reduction in mild to moderate depression [41]. It is important to note that excess homocysteine affects a person’s nervous system by directly damaging the neurons, including oxidative stress [42].

One of the limitations of the present study was that it coincided with the increase in the prevalence of COVID-19 in Iran, which might have indirectly affected the results of the study. The authors of this study suggest that researchers in other parts of the world conduct more studies to find out the mechanism of saffron's effect and provide more guarantee for its happy effects.

## Conclusion

Saffron herbal tea increased the mean happiness score in the intervention group and a significant difference was observed between the intervention and control groups. Therefore, it is suggested that people should consult with their doctor, and in case he/she approves, use complementary therapies such as saffron herbal teas along with other drugs prescribed by the doctor. On the other hand, other researchers are recommended to conduct more clinical studies in different population groups to establish the role of saffron herbal tea in reducing depression and increasing happiness scores.

## Data Availability

The data used and analyzed in this study are available from the corresponding author on reasonable request.
